# Association Between Neutrophil Percentage-to-Albumin Ratio (NPAR) and the Prognosis of Non-Small-Cell Lung Cancer

**DOI:** 10.3390/cancers18081283

**Published:** 2026-04-18

**Authors:** Xin Ye, Yi Liu, Fanjie Meng, Bin Hu, Hui Li

**Affiliations:** Department of Thoracic Surgery, Beijing Institute of Respiratory Medicine and Beijing Chao-Yang Hospital, Capital Medical University, Beijing 100020, China; x_007william@126.com (X.Y.); drliuyi1987@hotmail.com (Y.L.); mfjwill99@gmail.com (F.M.)

**Keywords:** neutrophil percentage-to-albumin ratio, non-small-cell lung cancer, prognosis, overall survival, nomogram, decision curve analysis

## Abstract

**Objective**: This study investigates the prognostic value and clinical utility of the neutrophil percentage-to-albumin ratio (NPAR) in patients with resected non-small-cell lung cancer (NSCLC). **Methods**: We retrospectively included 335 patients with NSCLC who underwent lung resection at our institution between January 2017 and October 2018. Optimal cutoffs for preoperative and postoperative day 1 (D1) NPAR were determined using X-tile (version 3.6.1; Yale University, New Haven, CT, USA) to define high and low groups. Overall survival (OS) was evaluated using Kaplan–Meier analysis and Cox proportional hazards models. A perioperative NPAR trajectory (low–low, low–high, high–low, high–high) was constructed to characterize dynamic risk patterns. To mitigate potential bias associated with postoperative measurements, a D1 landmark analysis was performed. A nomogram was developed based on the multivariable model and assessed by calibration at 1, 3, and 5 years. Incremental clinical value beyond TNM stage and surgical approach was evaluated using decision curve analysis (DCA), as well as by 5-year continuous net reclassification improvement (NRI) and integrated discrimination improvement (IDI). **Results**: The optimal cutoffs for preoperative and postoperative D1 NPAR were 14.5 and 23.1, respectively. In univariate analyses, sex, smoking history, preoperative NPAR, postoperative D1 NPAR, pathologic type, TNM stage, surgical approach, and adjuvant therapy were associated with OS (all *p* < 0.01). In multivariable Cox regression, high preoperative NPAR (HR 1.896, 95% CI 1.135–3.168; *p* = 0.014) and high postoperative D1 NPAR (HR 1.905, 95% CI 1.097–3.305; *p* = 0.014) were independent risk factors, along with TNM stage (Stage II: HR 2.824, 95% CI 1.209–6.595; *p* = 0.016; Stage III: HR 9.470, 95% CI 4.935–18.171; *p* < 0.001) and open surgery (HR 2.350, 95% CI 1.341–4.117; *p* = 0.003). Trajectory analysis further stratified risk, with the high–high group showing the poorest survival (adjusted HR 3.48, 95% CI 1.43–8.47; *p* = 0.006). The association of postoperative NPAR persisted in the D1 landmark analysis (HR 1.836, 95% CI 1.071–3.148; *p* = 0.027). Adding NPAR to TNM stage and surgical approach improved 5-year risk reclassification (continuous NRI 0.377, 95% CI 0.094–0.659; IDI 0.028, 95% CI −0.002–0.054) and increased net benefit on DCA. The nomogram demonstrated acceptable calibration at 1, 3, and 5 years. **Conclusions**: This study demonstrates that NPAR serves as an independent prognostic marker for long-term outcomes in patients with NSCLC. The use of NPAR offers clinicians a comprehensive and precise tool for assessing patient prognosis.

## Simple Summary

This retrospective study included 335 patients with resected non-small-cell lung cancer to assess the prognostic value of the neutrophil percentage-to-albumin ratio (NPAR). The optimal cutoffs were 14.5 for preoperative NPAR and 23.1 for NPAR on postoperative day 1. Multivariate analysis confirmed high preoperative and postoperative NPAR as independent predictors of poor overall survival. Patients with a persistently high perioperative NPAR trajectory exhibited the poorest clinical outcomes. Incorporating NPAR into a model with TNM stage and surgical approach improved risk stratification. A prognostic nomogram combining NPAR and clinical factors demonstrated robust predictive accuracy. NPAR, a readily available blood biomarker, can effectively refine prognosis evaluations for postoperative non-small-cell lung cancer patients.

## 1. Introduction

Nowadays, lung cancer is still a major problem worldwide [1]. Non-small-cell lung cancer (NSCLC) is the most common histological subtype, accounting for more than 80% of all lung cancer cases. Surgical resection remains the primary treatment modality for patients with early-stage NSCLC [2]. Nevertheless, postoperative outcomes after resection remain suboptimal [3,4]. Importantly, patients with similar TNM stages and histologic profiles may experience markedly different trajectories after surgery, underscoring the need for complementary prognostic indicators that are objective, readily available, and clinically actionable to refine postoperative risk stratification and guide surveillance intensity and perioperative management.

A growing body of evidence indicates that chronic inflammation and malnutrition are not merely epiphenomena but active contributors to tumor initiation, progression, and prognosis [5,6]. Neutrophils, as key effectors of innate immunity, can reflect an activated systemic inflammatory state and have been associated with tumor-promoting processes such as cytokine release, immunosuppressive signaling, and facilitation of tumor invasion and metastasis [7,8]. In parallel, serum albumin is a classical marker of nutritional reserve and systemic catabolism; hypoalbuminemia in cancer patients may indicate impaired nutritional and immune function and reduced physiologic resilience [9]. Therefore, indices integrating inflammatory and nutritional dimensions may provide a more holistic representation of host status than single laboratory parameters. In NSCLC, increasing evidence has shown that inflammation- and nutrition-based biomarkers are significantly associated with survival across both surgical and systemic treatment settings, including albumin-related composite indices and neutrophil-derived inflammatory scores [10,11,12]. Large cohort and retrospective studies have further supported the prognostic relevance of host inflammatory–nutritional status in lung cancer, suggesting that such biomarkers may complement conventional tumor-centered staging systems.

The neutrophil percentage-to-albumin ratio (NPAR), calculated as the neutrophil percentage divided by serum albumin level, integrates inflammatory activation and nutritional reserve into a single metric. Prior studies have reported the prognostic value of NPAR in multiple clinical settings, including cardiovascular disease, acute kidney injury, cardiogenic shock, liver cirrhosis, diabetes mellitus, pancreatic cancer, and bladder cancer [13,14,15,16,17,18,19,20]. In lung cancer, although direct evidence regarding NPAR remains limited, several related studies provide important support for its potential relevance. Previous studies have shown that neutrophil- and albumin-based composite markers, such as platelet-to-albumin ratio, albumin combined with neutrophil-to-lymphocyte ratio, and other inflammatory–nutritional scores, are independently associated with survival outcomes in NSCLC [21]. In addition, one previous report suggested that the neutrophil-to-albumin ratio was associated with NSCLC stage, further supporting the biological and clinical rationale for investigating NPAR in this disease setting [22]. Studies in advanced NSCLC treated with pembrolizumab-, nivolumab-, or bevacizumab-based regimens have also consistently shown that low albumin and elevated neutrophil-related parameters are associated with worse survival, indicating that this host-response axis may remain relevant across different therapeutic contexts [22,23,24,25]. However, the evidence for resectable NSCLC remains limited and inconsistent, and several clinically relevant questions remain unanswered. First, most studies focus on a single-time-point assessment, whereas perioperative care is intrinsically dynamic: surgical stress, inflammatory response, and nutritional catabolism may jointly reshape early postoperative physiology. Thus, whether early postoperative NPAR—particularly on postoperative day 1—captures additional prognostic information beyond preoperative status warrants evaluation. Second, beyond absolute values, the perioperative pattern may be clinically informative. For example, a persistently elevated profile from preoperative to early postoperative assessment may represent a vulnerable host-response phenotype, whereas normalization or transient elevation may carry different implications. Third, even if an association exists, its clinical relevance depends on whether incorporating perioperative NPAR improves risk stratification beyond conventional factors (e.g., TNM stage and surgical approach) and provides measurable net benefit for decision-making. Therefore, this study aimed to investigate the prognostic value of perioperative NPAR, explore the clinical significance of the perioperative NPAR trajectory, and evaluate its incremental clinical utility for postoperative risk stratification beyond conventional factors in patients with resectable NSCLC.

## 2. Materials and Methods

### 2.1. Study Population

This was a single-center retrospective study that included clinical data from 335 patients with NSCLC who underwent surgical treatment at the Beijing Chao-Yang Hospital, Capital Medical University, between January 2017 and October 2018. The inclusion criteria were: (I) histopathological confirmation of NSCLC; (II) complete R0 resection, defined as microscopically negative margins of the bronchus, arteries, veins, peribronchial tissues, and adjacent structures. The exclusion criteria were: (I) receipt of neoadjuvant therapy prior to surgery; (II) history of other malignancies; (III) history of hematologic or infectious diseases; (IV) incomplete clinical or follow-up data. The study was conducted in accordance with the Declaration of Helsinki (revised in 2013). Ethical approval was obtained from the Institutional Review Board of Beijing Chao-Yang Hospital (No. 2024-ke-28), and individual consent for this retrospective analysis was waived.

### 2.2. Data Collection and Follow-Up

Clinical data were collected through the electronic medical record system, including patient age, sex, height, weight, smoking history, pathological subtype, tumor-node-metastasis (TNM) stage, and surgery-related information (surgical type, surgical approach, and operation time). We performed a sensitivity analysis using albumin < 4.2 g/dL (<42 g/L) to facilitate clinical interpretability and external comparability. To enhance comparability between groups, all laboratory measurements used for NPAR calculation were obtained at predefined and consistent perioperative time points for all patients. To ensure data consistency and standardization, all blood samples were collected under fasting conditions between 6:00 AM and 8:00 AM according to standardized laboratory protocols at our institution. Fasting peripheral venous blood samples obtained within 24 h of admission and on the first postoperative day were analyzed. The neutrophil percentage-to-albumin ratio (NPAR) was calculated as neutrophil percentage (%) × 100/albumin concentration (g/dL). The optimal cutoff value of NPAR was determined using X-tile software (version 3.6.1; Yale University, New Haven, CT, USA). In this study, the optimal thresholds for preoperative NPAR and postoperative day 1 NPAR were 14.5 and 23.1, respectively. All pathological diagnoses, staging evaluations, surgical eligibility assessments, and perioperative laboratory tests were performed according to routine institutional standards at the same center, thereby reducing inter-institutional variability. Baseline clinicopathological characteristics were compared between the low- and high-NPAR groups at each time point to assess group comparability after stratification. Preoperative serum albumin was additionally analyzed as an individual prognostic biomarker.

### 2.3. Definition of NPAR and Perioperative Dynamics

Neutrophil percentage-to-albumin ratio (NPAR) was calculated using routine blood test data, integrating systemic inflammatory activity and nutritional reserve. NPAR was computed preoperatively and on postoperative day 1 (D1). The optimal cutoffs for preoperative NPAR and postoperative D1 NPAR were determined using X-tile, and patients were categorized into low- and high-NPAR groups accordingly. To capture perioperative dynamics, patients were further classified into four trajectory groups: low–low, low–high, high–low, and high–high, based on the preoperative and postoperative cutoffs. In addition, the perioperative change was explored as a continuous variable (ΔNPAR = D1 NPAR − preoperative NPAR) to assess whether short-term magnitude of change provided incremental prognostic information.

Patient follow-up was conducted through telephone interviews and outpatient visits, during which information on postoperative adjuvant therapy was also documented. In this study, overall survival (OS) was defined as the time from the date of surgery to the date of last follow-up or death. The median follow-up duration was 62 months (interquartile range (IQR): 58–70 months), during which 73 death events were recorded.

### 2.4. Statistical Analysis

Continuous variables were compared between groups using the independent samples t-test or the Mann–Whitney U test. Categorical variables were analyzed using the chi-square test or Fisher’s exact test. Univariate and multivariate Cox proportional hazards regression analyses were performed to identify independent prognostic factors associated with OS. Based on the independent risk factors, a nomogram was developed to predict long-term survival in NSCLC patients. Univariate and multivariate Cox proportional hazards regression models were applied to identify prognostic factors for OS (Table 1). The primary multivariable model included preoperative NPAR (high vs. low), postoperative D1 NPAR (high vs. low), TNM stage, and surgical approach, consistent with clinical relevance and univariate screening. Univariate and multivariable Cox proportional hazards regression analyses were performed to identify independent prognostic factors for OS (Table 2). To mitigate potential bias that postoperative biomarkers might reflect early perioperative events (reverse causality), a D1 landmark analysis was performed: deaths occurring before D1 were excluded, and follow-up time was redefined from D1 onward (Table 3A). A prognostic nomogram was constructed from the multivariable Cox model. Model calibration was assessed using 1-, 3-, and 5-year calibration curves (Panels A–C), with observed OS estimated by Kaplan–Meier methods within deciles of predicted survival; IPCW Brier scores and calibration slope/intercept were additionally reported. The incremental clinical utility of adding NPAR to a base mod el (TNM stage + surgical approach) was quantified via decision curve analysis (DCA) at 5 years and continuous NRI/IDI at 5 years with bootstrap confidence intervals (Table 3B). To examine whether the prognostic value of NPAR was independent of albumin, additional Cox models were constructed: (i) albumin alone (continuous and dichotomized at 4.2 g/dL), (ii) NPAR alone, and (iii) NPAR jointly modeled with albumin. Trajectory group effects were evaluated using adjusted Cox models with low–low as the reference (Table 3C). In addition, neutrophil fraction and albumin were jointly modeled to assess the independent contributions of inflammation and nutrition components (Table 3D). Calibration curves were plotted to evaluate the agreement between predicted and observed survival probabilities, and the predictive performance of the nomogram was assessed using receiver operating characteristic (ROC) curve analysis. A two-sided *p*-value of <0.05 was considered statistically significant. All statistical analyses were performed using SPSS (version 26.0; IBM Corp., Armonk, NY, USA) and R (version 4.4.1; R Foundation for Statistical Computing, Vienna, Austria).

## 3. Results

### 3.1. Correlation Between NPAR and Clinical Features

Based on the optimal cutoff values of preoperative and postoperative NPAR (14.5 and 23.1, respectively), patients were stratified to further analyze their clinical characteristics. For preoperative NPAR, patients were divided into a low-NPAR group (NPAR ≤ 14.5, *n* = 188) and a high-NPAR group (NPAR > 14.5, *n* = 147). For postoperative NPAR, patients were categorized into a low-NPAR group (NPAR ≤ 23.1, *n* = 138) and a high-NPAR group (NPAR > 23.1, *n* = 197) (Table 4). In the preoperative NPAR groups, significant differences were observed in age (*p* = 0.003), gender (*p* = 0.001), pathological type (*p* = 0.002), TNM stage (*p* = 0.011), surgical approach (*p* < 0.001), and operation time (*p* = 0.001). In the postoperative NPAR groups, patients differed significantly in age (*p* = 0.007), BMI (*p* < 0.001), pathological type (*p* = 0.004), surgical type (*p* = 0.046), surgical approach (*p* = 0.046), and operation time (*p* < 0.001) (Table 4).

Because BMI, operative time, and surgical approach differed significantly between the high- and low-NPAR groups (Table 1 and Table 4), we performed a sensitivity analysis by additionally adjusting for BMI and operative time in the primary Cox model. After additional adjustment, preoperative NPAR remained statistically significant (HR 1.983, 95% CI 1.210–3.251; *p* = 0.007), whereas the association for postoperative D1 NPAR was attenuated but directionally consistent (HR 1.655, 95% CI 0.928–2.949; *p* = 0.088). In this sensitivity model, BMI (HR 0.989, 95% CI 0.908–1.076; *p* = 0.796) and operative time (HR 1.006, 95% CI 0.965–1.049; *p* = 0.762) were not independently associated with OS.

Taken together, the observed differences in clinicopathological characteristics across NPAR strata indicate that perioperative NPAR is not randomly distributed but, rather, reflects meaningful variation in patient and disease status.

### 3.2. Univariate and Multivariate Cox Regression Analyses

In the univariate Cox regression analysis, several factors were significantly associated with the prognosis of NSCLC patients, including gender (*p* < 0.001), smoking history (*p* = 0.001), preoperative NPAR (*p* < 0.001), postoperative NPAR (*p* = 0.002), pathological type (*p* < 0.001), TNM stage (*p* < 0.001), surgical approach (*p* < 0.001), and adjuvant therapy (*p* < 0.001). These variables were subsequently included in the multivariate Cox regression analysis. The results revealed that preoperative NPAR (HR: 1.896, 95% CI: 1.135–3.168, *p* = 0.014), postoperative NPAR (HR: 1.905, 95% CI: 1.097–3.305, *p* = 0.014), TNM stage (Stage II: HR: 2.824, 95% CI: 1.209–6.595, *p* = 0.016; Stage III: HR: 9.470, 95% CI: 4.935–18.171, *p* < 0.001), and surgical approach (HR: 2.350, 95% CI: 1.341–4.117, *p* = 0.003) were independent prognostic factors for long-term survival in NSCLC patients (Table 2).

**Figure 1:**
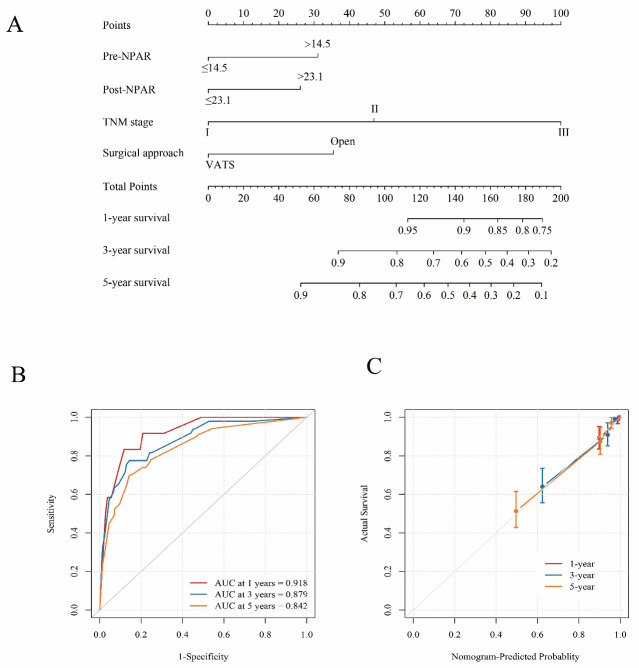
Development and performance of the prognostic nomogram incorporating perioperative NPAR. (**A**) Nomogram for predicting 1-, 3-, and 5-year overall survival (OS) in resected NSCLC, incorporating preoperative NPAR (cutoff 14.5), postoperative day 1 (D1) NPAR (cutoff 23.1), TNM stage, and surgical approach (VATS vs. open). Points assigned to each predictor are summed to obtain total points, which correspond to estimated survival probabilities at 1, 3, and 5 years. (**B**) Time-dependent ROC curves for the nomogram at 1, 3, and 5 years, showing discrimination performance (AUCs displayed in the panel). (**C**) Calibration plot of the nomogram for 1-, 3-, and 5-year OS, comparing nomogram-predicted probabilities with observed outcomes; the diagonal line indicates perfect agreement.

Kaplan–Meier survival curves (Figure 1) further illustrated the relationship between these independent risk factors and long-term survival. The results demonstrated significantly reduced survival in patients with high preoperative NPAR (log-rank *p* < 0.001), high postoperative NPAR (log-rank *p* = 0.001), thoracotomy (log-rank *p* < 0.001), and those classified as TNM stage II and III (log-rank *p* < 0.001). BMI and operation time were not independently associated with OS in this sensitivity model.

These findings indicate that NPAR retains prognostic significance at both preoperative and postoperative time points, highlighting the value of perioperative inflammatory–nutritional status for long-term outcome assessment in NSCLC.

### 3.3. Construction of a Nomogram Based on Independent Prognostic Factors

**Figure 2:**
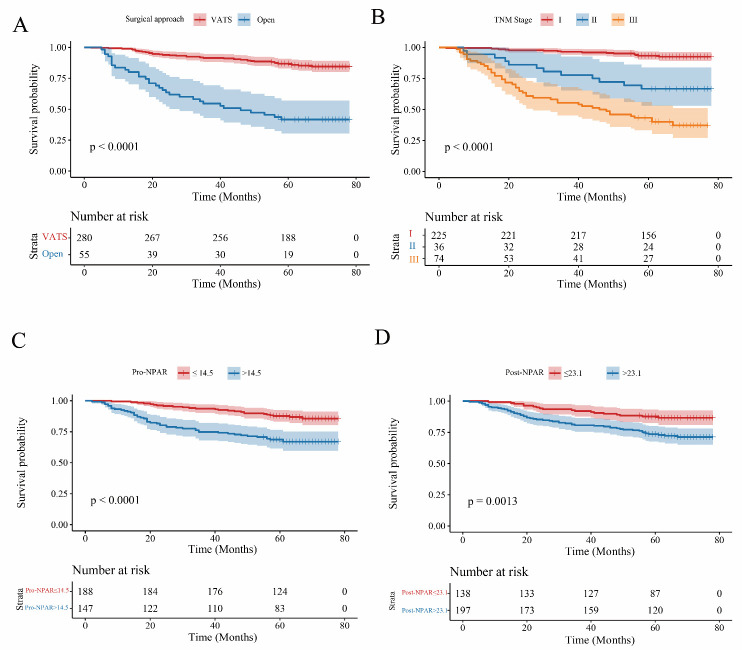
Kaplan–Meier overall survival curves stratified by clinicopathological factors and perioperative NPAR. Kaplan–Meier curves for OS with shaded 95% confidence intervals; tick marks (“+”) indicate censored observations. Numbers at risk are shown below each plot; *p*-values are from log-rank tests. (**A**) OS by surgical approach (VATS vs. open). (**B**) OS by TNM stage (I–III). (**C**) OS by preoperative NPAR group (low ≤ 14.5 vs. high >14.5). (**D**) OS by postoperative D1 NPAR group (low ≤ 23.1 vs. high > 23.1).

Based on the independent prognostic factors identified through Cox regression analysis, a nomogram model was constructed to predict 1-, 3-, and 5-year OS in NSCLC patients (Figure 2). The concordance index (C-index) of the nomogram was 0.855 (95% CI: 0.744–0.922), indicating a high level of predictive accuracy. In addition, ROC curve analysis demonstrated that the areas under the curve (AUC) for predicting 1-, 3-, and 5-year OS were 0.918, 0.879, and 0.842, respectively (Figure 1C).

These results suggest that incorporating perioperative NPAR into a multivariable nomogram may improve individualized risk estimation beyond conventional clinicopathological assessment alone.

### 3.4. Dynamic Trajectory, Landmark Robustness, Incremental Value, and Model Calibration

**Figure 3:**
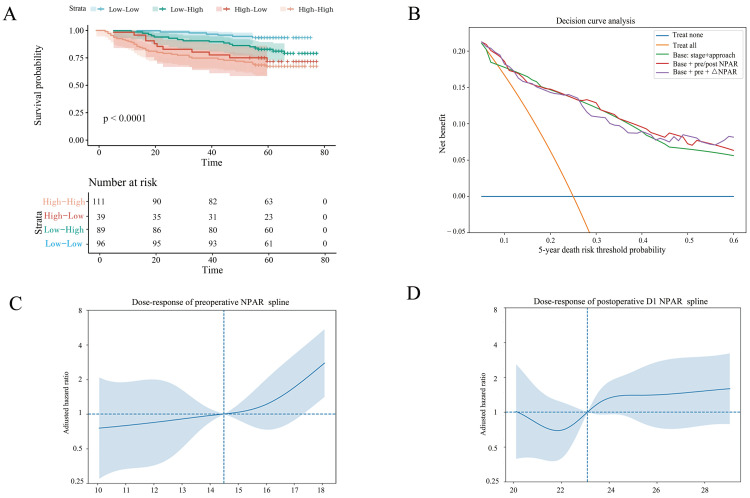
Dynamic perioperative NPAR trajectory, decision-curve analysis, and dose–response relationships. (**A**) Kaplan–Meier OS curves according to perioperative NPAR trajectory groups: low–low, low–high, high–low, and high–high (defined by preoperative cutoff 14.5 and postoperative D1 cutoff 23.1). Shaded areas denote 95% confidence intervals; “+” indicates censoring; numbers at risk are shown below. (**B**) Decision curve analysis (5-year horizon) comparing net benefit across threshold probabilities for different models, including treat-none and treat-all strategies, a base model (TNM stage + surgical approach), and models augmented with perioperative NPAR information (as labeled in the panel). (**C**) Dose–response relationship between preoperative NPAR and adjusted hazard ratio for OS estimated using a spline-based Cox model; the curve represents adjusted HR and the shaded band indicates 95% confidence interval (with the likelihood ratio test *p*-value for adding the spline term shown). (**D**) Dose–response relationship between postoperative D1 NPAR and adjusted hazard ratio for OS estimated using a spline-based Cox model; shaded band indicates 95% confidence interval.

Trajectory-based stratification yielded marked risk separation (Figure 3A). Compared with the low–low reference group, the high–high trajectory exhibited the worst prognosis (adjusted HR 3.48, 95% CI 1.43–8.47, *p* = 0.006; Table 3C), supporting the clinical importance of persistent perioperative elevation (Table 3A). By contrast, modeling perioperative change as a continuous measure showed that ΔNPAR (D1 − pre) did not independently predict OS after accounting for baseline NPAR and clinicopathological factors (per 1 SD: HR 1.103, 95% CI 0.870–1.398, *p* = 0.417; *p* for quartile trend = 0.310), suggesting that persistent elevation (trajectory status) rather than short-term magnitude of change is more informative for long-term prognosis.

In the D1 landmark analysis, high postoperative D1 NPAR remained significantly associated with subsequent mortality after adjustment (HR 1.836, 95% CI 1.071–3.148, *p* = 0.027), supporting the robustness of the postoperative association when follow-up was re-anchored from D1 onward (Table 3A). Adding NPAR to a base model of TNM stage and surgical approach improved 5-year risk reclassification (continuous NRI 0.377, 95% CI 0.094–0.659; IDI 0.028, 95% CI −0.002–0.054) and yielded higher net benefit across a range of clinically relevant threshold probabilities on DCA at 5 years (Figure 3B and Table 3B). Spline-based analyses did not indicate significant departures from linearity for either preoperative NPAR (*p* for non-linearity = 0.422) or postoperative D1 NPAR (*p* for non-linearity = 0.747; Figure 3C,D).

**Figure 4:**
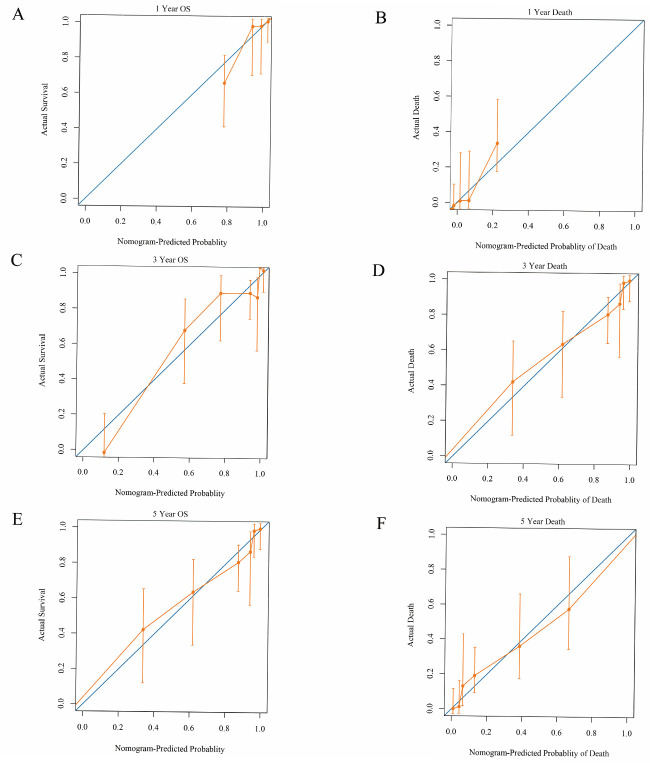
Calibration curves of the nomogram for 1-, 3-, and 5-year outcomes. Calibration curves comparing predicted probabilities from the nomogram with observed probabilities estimated within risk groups; the diagonal line indicates perfect calibration. (**A**–**C**) Calibration for probability of death by 1, 3, and 5 years, respectively (x-axis: predicted probability of death; y-axis: observed probability of death). (**D**–**F**) Calibration for overall survival (OS) probability at 1, 3, and 5 years, respectively (x-axis: predicted OS probability; y-axis: observed OS probability).

The nomogram derived from the multivariable Cox model showed acceptable calibration at 1, 3, and 5 years (Calibration Panels A–C, Figure 4A–C). IPCW Brier scores were 0.0287, 0.0809, and 0.1111 at 1, 3, and 5 years, respectively, with low decile-based mean absolute calibration errors (0.019, 0.054, and 0.027, Figure 4D–F), indicating reasonable agreement between predicted and observed OS (Table 5).

Collectively, these additional analyses support the robustness, incremental prognostic value, and clinical applicability of perioperative NPAR, indicating that persistent elevation rather than short-term fluctuation carries greater relevance for long-term survival prediction in resected NSCLC.

## 4. Discussion

In this retrospective cohort of resected NSCLC, we observed that both preoperative and postoperative NPAR were independently associated with overall survival, with higher values indicating an unfavorable prognosis. Given that NPAR can be derived directly from routine perioperative blood tests, it may represent a practical biomarker that can be integrated into everyday clinical workflows. In this context, NPAR-based risk stratification could assist clinicians in identifying patients who may benefit from closer follow-up and more individualized postoperative management after curative-intent surgery.

Preoperative serum albumin has been recognized as a prognostic marker in resected NSCLC. Miura et al. reported that an albumin cutoff of 4.2 g/dL could stratify both overall and recurrence-free survival in a surgical NSCLC cohort, and the prognostic effect varied across underlying lung conditions (normal lung, emphysema, and pulmonary fibrosis) [26]. Consistent with this literature, we observed that low preoperative albumin (<4.2 g/dL) was associated with poorer OS in our cohort. Importantly, when albumin and NPAR were modeled jointly, preoperative NPAR remained significant, whereas albumin was attenuated, suggesting that the prognostic information carried by NPAR is not merely a surrogate of hypoalbuminemia but reflects the combined inflammatory–nutritional phenotype.

Although NPAR has not been extensively studied for lung cancer, it has not been entirely unexplored. A previous report suggested that a neutrophil-to-albumin ratio was associated with NSCLC stage [22], while several related inflammation–nutritional indices incorporating neutrophil- and albumin-based parameters have also shown prognostic relevance in NSCLC, including albumin combined with neutrophil-derived markers [27], platelet-to-albumin ratio [28], and other composite inflammatory–nutritional scores [12]. In advanced NSCLC, studies of bevacizumab-, pembrolizumab-, and nivolumab-treated cohorts have further supported the prognostic importance of the neutrophil/albumin axis [23,25,29]. However, prior evidence has been limited, indirect, and heterogeneous, and the role of perioperative NPAR in resectable NSCLC has remained unclear. In this context, our study extends the existing literature by specifically focusing on resectable disease and by evaluating perioperative timing, trajectory pattern, and incremental prognostic value beyond conventional clinicopathological factors.

In a retrospective analysis of 145 unresectable pancreatic adenocarcinoma, the NPAR was confirmed as an independent survival predictor [18]. Similarly, studies have shown that elevated NPAR correlates with poor survival outcomes in bladder cancer patients [17], as well as in colorectal adenocarcinoma [30]. In oral squamous cell carcinoma, poorer survival has been associated with an increased NPAR, which may arise from a higher circulating neutrophil burden and lower albumin levels [31]. Collectively, these observations across tumor types are consistent with the notion that NPAR reflects host-related biological states relevant to cancer progression. From a biological and analytical standpoint, NPAR can be regarded as a composite indicator capturing two clinically meaningful dimensions—systemic inflammation and nutritional reserve. Compared with using neutrophil measures or albumin alone, an integrated ratio may provide a more stable representation of the patient’s overall condition and may better reflect the balance between inflammatory activation and nutritional impairment [11]. Because neutrophil percentage and serum albumin can each fluctuate due to multiple physiological and perioperative influences, combining them into a single index may reduce reliance on any single component and improve robustness in real-world settings.

Despite the accumulating evidence for its prognostic value, the mechanisms linking NPAR to outcomes in NSCLC remain incompletely understood. NPAR, calculated from neutrophil percentage and serum albumin, mirrors the inflammatory and nutritional milieu of the host—two factors that are closely intertwined with cancer progression and metastatic potential [32]. Prior studies have highlighted a strong relationship between systemic inflammatory responses and carcinogenesis [33,34]. Neutrophils may facilitate tumor progression through several mechanisms, including promotion of angiogenesis and suppression of T-cell-mediated antitumor immunity [35,36]. In support of this concept, one study of 309 patients reported that higher neutrophil counts were associated with poorer overall and disease-free survival [37]. On the nutritional side, Lim et al. found that lower pretreatment albumin levels correlated with worse OS and DFS in 338 patients treated for head and neck cancer [38]. Malnutrition may compromise antitumor immunity and increase vulnerability to infection and postoperative complications [27,28,39,40]. Furthermore, heavier tumor burden may coincide with enhanced systemic inflammation, neutrophil recruitment, and greater albumin depletion, which together could partially explain why a higher NPAR tracks with adverse survival in malignancy [41]. Nevertheless, these hypotheses require further experimental and clinical investigation, particularly in resectable NSCLC.

This study demonstrates that NPAR, a composite inflammation–nutrition marker, is independently associated with long-term survival in resected NSCLC when assessed both preoperatively and on postoperative day 1. The persistence of the postoperative association in a D1 landmark framework strengthens inference by reducing concern that postoperative NPAR merely reflects early perioperative events or reverse causation [42,43]. Moreover, the pronounced separation achieved by trajectory stratification—particularly the high–high phenotype—substantiates the hypothesis that persistent perioperative inflammatory–nutritional dysregulation represents a clinically meaningful host-response state linked to adverse outcomes [32,44]. In contrast, ΔNPAR (D1 − pre) did not confer independent prognostic information beyond baseline NPAR and core clinicopathological factors, suggesting that long-term risk is better captured by a state of sustained elevation rather than the short-term magnitude of perioperative change. To address this concern, we performed a sensitivity analysis adding BMI and operative time to the primary Cox model. After additional adjustment, the prognostic association of preoperative NPAR remained stable and statistically significant, supporting that preoperative NPAR captures risk information beyond body size and operative duration.

From a translational standpoint, incorporating NPAR into a conventional model (TNM stage and surgical approach) improved reclassification and net benefit at the 5-year horizon, suggesting potential utility for risk-adapted surveillance and perioperative decision support. Mechanistically, a high NPAR may reflect heightened innate inflammatory activity coupled with reduced albumin, indicating impaired nutritional reserve, systemic stress, and potentially compromised anti-tumor immunity factors that could influence recurrence and mortality after resection [33,34,45,46]. The findings also highlight a potential avenue for perioperative optimization strategies targeting inflammatory and nutritional status, particularly in patients with persistently high NPAR.

## 5. Conclusions

Several limitations should be acknowledged. First, this was a retrospective single-center study, and residual selection bias and unmeasured confounding could not be fully excluded despite multivariable adjustment. Second, NPAR is a non-specific host-response biomarker; both neutrophil percentage and serum albumin may be influenced by conditions unrelated to tumor biology, such as infection, liver or renal dysfunction, and perioperative supportive care, which may have affected the observed associations. Third, postoperative NPAR was measured on postoperative day 1, a time point strongly influenced by surgical trauma and early inflammatory stress and therefore should be interpreted as reflecting early postoperative host status rather than tumor-related biology alone. Finally, the cutoff values were derived from the present cohort, and no independent external validation cohort was available, which may limit the generalizability and immediate clinical applicability of our findings. Future multi-center prospective studies are needed to validate the robustness, transferability, and optimal clinical use of perioperative NPAR in resectable NSCLC. In conclusion, our study demonstrates that NPAR can serve as an independent predictor of long-term prognosis in patients with NSCLC. The application of NPAR offers clinicians a comprehensive and accurate tool for assessing patient outcomes.

## Figures and Tables

**Table 1:** Association between postoperative day 1 (D1) NPAR and patients’ clinicopathological characteristics.

Characteristic	Low NPAR (*n* = 138)	High NPAR (*n* = 197)	*p*-Value
Age, median (IQR), years	60 (55–64)	63 (55–68)	**0.007**
Gender, *n* (%)			0.088
Male	57 (41.3)	100 (50.8)	
Female	81 (58.7)	97 (49.2)	
BMI, median (IQR), kg/m^2^	24.60 (22.49–27.27)	23.33 (21.47–25.58)	**<0.001**
Smoking history, *n* (%)	47(34.1)	80 (40.6)	0.224
Pathologic type, *n* (%)			**0.004**
Adenocarcinoma	121 (87.7)	148 (75.1)	
Non-adenocarcinoma	17 (12.3)	49 (24.9)	
TNM stage			0.084
I	102 (73.9)	123 (62.4)	
II	11 (8.0)	25 (12.7)	
III	25 (18.1)	49 (24.9)	
Surgical type, *n* (%)			**0.046**
Lobe	121 (87.7)	185 (93.9)	
Seg/Wed	17 (12.3)	12 (6.1)	
Surgical approach, *n* (%)			**0.046**
VATS	122 (88.4)	158 (80.2)	
Open	16 (11.6)	39 (19.8)	
Adjuvant therapy, *n* (%)	51 (37.0)	72 (36.5)	0.939
Operation time, median (IQR), min	150 (120–180)	170 (143–210)	**<0.001**

NPAR, neutrophil percentage-to-lbumin ratio; IQR, interquartile range; BMI, body mass index; Lobe, lobectomy; Seg, segmentectomy; Wed, wedge resection; VATS, video-assisted thoracoscopic surgery. The bold values indicate the value of *p* < 0.05, which is statistically significant.

**Table 2:** Univariate and multivariate Cox regression analyses for overall survival.

Variables	Univariable Cox Regression		Multivariable Cox Regression	
	HR (95% CI)	*p*-Value	HR (95% CI)	*p*-Value
Age, years				
<60	1.000			
≥60	1.341 (0.834–2.156)	0.227		
Gender				
Male	1.000		1.000	
Female	0.387 (0.238–0.631)	**<0.001**	0.642 (0.314–1.312)	0.224
BMI, kg/m^2^				
<23.88	1.000			
≥23.88	0.811 (0.512–1.285)	0.372		
Smoking history				
No	1.000		1.000	
Yes	2.196 (1.385–3.483)	**0.001**	1.300 (0.674–2.508)	0.434
Pre-NPAR				
≤14.5	1.000		1.000	
>14.5	2.862 (1.764–4.642)	**<0.001**	1.896 (1.135–3.168)	**0.014**
Po-NPAR				
≤23.1	1.000		1.000	
>23.1	2.332 (1.369–3.972)	**0.002**	1.905 (1.097–3.305)	**0.022**
Pathologic type				
Adenocarcinoma	1.000		1.000	
Non-adenocarcinoma	2.821 (1.753–4.541)	**<0.001**	0.584 (0.310–1.104)	0.098
TNM stage				
I	1.000		1.000	
II	5.351 (2.530–11.317)	**<0.001**	2.824 (1.209–6.595)	**0.016**
III	12.857 (7.248–22.806)	**<0.001**	9.470 (4.935–18.171)	**<0.001**
Surgical type				
Seg/wed	1.000			
Lobe	2.312 (0.727–7.345)	0.155		
Surgical approach, *n* (%)				
VATS	1.000		1.000	
Open	5.736 (3.604–9.129)	**<0.001**	2.350 (1.341–4.117)	**0.003**
Adjuvant therapy, *n* (%)				
No	1.000		1.000	
Yes	2.723 (1.708–4.343)	**<0.001**	1.160 (0.690–1.950)	0.575

BMI, body mass index; NPAR, neutrophil percentage-to-lbumin ratio; pre-NPAR, preoperative neutrophil percentage-to-lbumin ratio; po-NPAR, postoperative neutrophil percentage-to-lbumin ratio; Lobe, lobectomy; Seg, Segmentectomy; Wed, wedge resection; VATS, video-assisted thoracoscopic surgery. The bold values indicate the value of *p* < 0.05, which is statistically significant.

**Table 3:** (**A**) Landmark Cox analysis anchored at postoperative day 1; (**B**) 5-year risk reclassification, continuous NRI and IDI; (**C**) adjusted Cox model for NPAR trajectory groups; (**D**) prognostic value of preoperative serum albumin and independence analyses.

**(A)**				
**Predictor**	**HR**	**95% CI_Low**	**95% CI_High**	***p***
Pre-op NPAR high (>14.5)	1.997	1.219	3.27	0.006
Post-op NPAR high (>23.1)	1.717	1.001	2.946	0.049
Pre-op NPAR (per 1 SD)	1.601	1.237	2.072	0
ΔNPAR (D1 − pre) (per 1 SD)	1.103	0.87	1.398	0.417
ΔNPAR quartile trend (per quartile)	1.118	0.901	1.388	0.31
Post-op NPAR high (>23.1)	1.836	1.071	3.148	0.027
Post-op NPAR (per 1 SD)	1.19	0.978	1.449	0.083
**(B)**				
**Comparison**	**NRI**	**95% CI**	**IDI**	**95% CI**
Base vs. Base + pre/post NPAR	0.377	0.094–0.659	0.028	−0.002–0.054
Base vs. Base + pre + ΔNPAR	0.309	0.036–0.570	0.026	−0.004–0.057
**(C)**				
**Trajectory group (pre/post)**	***n***	**Adjusted HR**	**95% CI**	***p*** **-Value**
Low–Low	96	1	Reference	—
Low–High	89	2.15	0.86–5.36	0.1
High–Low	39	2.43	0.93–6.34	0.069
High–High	111	3.48	1.43–8.47	0.006
**(D)**				
**Model**	**Variable**	**HR (95% CI)**		***p*** **-Value**
Univariate	Albumin < 42 g/L (4.2 g/dL)	2.596 (1.575–4.281)		<0.001
Adjusted (Stage + approach)	Albumin < 42 g/L	1.753 (1.037–2.961)		0.036
Adjusted (Stage + approach + NPAR)	Preoperative NPAR high (>14.5)	1.941 (1.175–3.205)		0.010
Adjusted (Stage + approach + NPAR)	Albumin < 42 g/L	1.534 (0.898–2.619)		0.117
Adjusted (Stage + approach + components)	Neutrophil fraction (per 1 SD)	1.403 (1.102–1.786)		0.006
Adjusted (Stage + approach + components)	Albumin (per 1 SD)	0.686 (0.526–0.895)		0.005

Base model = TNM stage + surgical approach. Predictions derived from Cox models. Outcome = 5-year death (complete-case at 60 months; censored before-60-months excluded). 95% CIs from bootstrap (800 resamples). Adjusted for TNM stage (II vs. I; III vs. I) and surgical approach (open vs. VATS). Reference = Low–Low.

**Table 4:** Association between preoperative NPAR and patients’ clinicopathological characteristics.

Characteristic	Low NPAR (*n* = 188)	High NPAR (*n* = 147)	*p*-Value
Age, median (IQR), years	60 (54–65)	63 (57–68)	**0.003**
Gender, *n* (%)			**0.001**
Male	73 (38.8)	84 (57.1)	
Female	115 (61.2)	63 (42.9)	
BMI, median (IQR), kg/m^2^	24.19 (22.18–26.67)	23.39 (21.80–25.71)	0.069
Smoking history, *n* (%)	64 (34.0)	63 (42.9)	0.099
Pathologic type, *n* (%)			**0.002**
Adenocarcinoma	162 (86.2)	107 (72.8)	
Non-adenocarcinoma	26 (13.8)	40 (27.2)	
TNM stage			**0.011**
I	139 (73.9)	86 (58.5)	
II	17 (9.0)	19 (12.9)	
III	32 (17.0)	42 (28.6)	
Surgical type, *n* (%)			0.776
Lobe	171 (91.0)	135 (91.8)	
Seg/wed	17 (9.0)	12 (8.2)	
Surgical approach, *n* (%)			**<0.001**
VATS	170 (90.4)	110 (74.8)	
Open	18 (9.6)	37 (25.2)	
Adjuvant therapy, *n* (%)	62 (33.0)	61 (41.5)	0.108
Operation time, median (IQR), min	150 (125–180)	170 (150–200)	**0.001**

NPAR, neutrophil percentage-to-lbumin ratio; IQR, interquartile range; BMI, body mass index; Lobe, lobectomy; Seg, segmentectomy; Wed, wedge resection; VATS, video-assisted thoracoscopic surgery. The bold values indicate the value of *p* < 0.05, which is statistically significant.

**Table 5:** Calibration metrics panels ABC.

Panel	Horizon	Brier (IPCW; Survival)	Cal Intercept (IPCW-Logistic)	Cal Slope (IPCW-Logistic)	Mean Abs Cal Error (Deciles)
A	1-year	0.0287	1.026	1.464	0.023
B	3-year	0.0809	0.13	1.089	0.044
C	5-year	0.1111	−0.232	0.892	0.034

## Data Availability

Data supporting the findings of this study can be obtained from the corresponding authors upon reasonable request.
